# Case Report: Exogenous insulin antibody syndrome complicated with chronic renal failure and long-term history of type 2 diabetes: report of two cases

**DOI:** 10.3389/fendo.2025.1676062

**Published:** 2025-10-29

**Authors:** Zhiwei Hu, Xiaozhu Huang, Jun Pan, Hua Dong

**Affiliations:** ^1^ Department of Endocrinology, The First People's Hospital of Jiashan, Jiashan Hospital Affiliated of Jiaxing University, Jiaxing, Zhejiang, China; ^2^ Department of Emergency Medicine, The First People’s Hospital of Jiashan, Jiashan Hospital Affiliated of Jiaxing University, Jiaxing, Zhejiang, China

**Keywords:** exogenous insulin antibody syndrome, hyperinsulinemia, insulin autoantibodies, chronic renal failure, dorzagliatin

## Abstract

**Background:**

Insulin autoimmune syndrome (IAS), a rare condition caused by an endogenous insulin-induced autoimmune reaction, is characterized by recurrent hypoglycemic episodes, positive insulin autoantibodies (IAAs), and high serum insulin levels. However, recent studies have demonstrated that exogenous insulin administration can also lead to similar clinical manifestations and have proposed the concept of non-classical IAS. This article reports two cases of exogenous insulin antibody syndrome (EIAS), analyzes their clinical features, and describes our therapeutic approach.

**Case presentation:**

The first patient was an 83-year-old male with a 15-year history of type 2 diabetes, while the second patient was an 86-year-old male with a 20-year history of type 2 diabetes. Both patients had a history of exogenous insulin use and chronic renal failure. On admission, they exhibited alternating episodes of hypoglycemia and hyperglycemia. Laboratory tests revealed hyperinsulinemia (insulin >600.00 μIU/ml in both patients), a dissociation phenomenon between blood insulin and C-peptide levels, and positive IAAs.

**Conclusion:**

In both patients, glycemic fluctuations resolved following insulin discontinuation and the initiation of dorzagliatin, confirming the diagnosis of EIAS. In type 2 diabetic patients with unexplained hypoglycemic and hyperglycemic episodes with a history of exogenous insulin use, pancreatic function and the autoimmune antibody spectrum should be comprehensively evaluated to rule out EIAS and provide an accurate diagnosis and guide treatment strategies.

## Introduction

Insulin autoimmune syndrome (IAS) is a rare disease that is frequently overlooked or misdiagnosed in clinical settings. It was first reported by the Japanese scholar Hirata in 1970; hence, it is also referred to as Hirata’s disease (1). Since then, cases have been identified in numerous countries. Indeed, 795 cases of IAS have been documented worldwide, with the highest prevalence in Japan and China (2) and relatively fewer reports among Caucasian populations (3). Hyperinsulinemia, positive insulin antibodies, and fluctuations in glycemic levels without the use of insulin are hallmarks of IAS. Additionally, recent studies have described that hypoglycemic episodes caused by exogenous insulin in diabetic patients are similar to those of IAS. As a result, this condition is also referred to as exogenous insulin antibody syndrome (EIAS), representing a non-classical form of IAS (4). A recent study (5) found 50 confirmed cases of EIAS, with a median age of symptom presentation of 70 years (range: 15–85 years), a slight male predominance (M 68%; F 32%), and 68% of these patients having intractable hypoglycemia. Both patients with T1D or T2D might have EIAS under insulin therapy. Here, we report two typical cases of EIAS with chronic renal failure and a long-term history of type 2 diabetes, contributing to the diagnosis and management of this condition.

## Case 1

**Figure 1:**
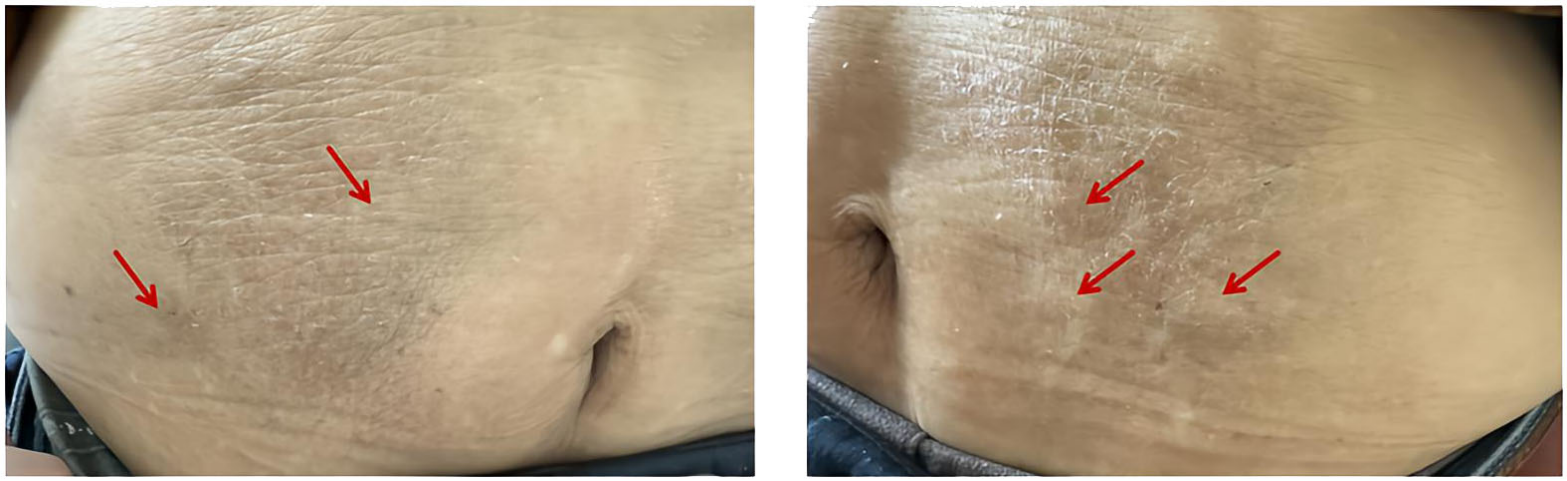
Numerous subcutaneous nodules surrounding the umbilical region, as indicated by red arrows.

An 83-year-old male patient with a 15-year history of type 2 diabetes and a 7-year history of diabetic kidney disease (DKD) was admitted to our hospital. At the initial onset of diabetes, he was treated with unspecified oral hypoglycemic medications. One year prior to admission, insulin aspart 30 injection (Novo Nordisk A/S, Tianjin, China) was initiated at a dose of 16 units before breakfast and 10 units before dinner, in combination with oral linagliptin 5 mg once daily. Notably, the patient reported infrequent replacement of insulin injection needles. One week before admission, he experienced fatigue and dry mouth. As a result, he attended a local hospital. Laboratory tests revealed a fasting blood glucose (FBG) level of 11.45 mmol/L, serum creatinine of 218 umol/L, and glycated hemoglobin (HbA1c) level of 14.4%. He was subsequently transferred to our department for further assessment. Upon admission, his vital signs were as follows: temperature: 36.6°C, pulse: 86 beats per minute, blood pressure: 143/86 mmHg. He was conscious, alert, and oriented. Cardiovascular examination unveiled a regular heart rhythm. Moreover, the abdomen was soft, with multiple subcutaneous nodules noted around the umbilical region (**Figure 1**). He had a past medical history of chronic kidney disease, hypertension, heart failure, and cerebral infarction. He was currently receiving atorvastatin calcium, amlodipine, isosorbide mononitrate, febuxostat, and spironolactone.

Laboratory tests demonstrated the 24h urinary protein excretion was 457.5 mg/24h, hemoglobin was 89 g/L, and creatinine was 224 μmol/L, yielding an eGFR of 23.06 ml/(min/1.73 m²). Thyroid function, cortisol, and adrenocorticotropic hormone levels were within normal ranges.

Routine scans of the head, abdomen, and chest revealed bilateral frontal-parietal and periventricular low-density foci, senile brain changes, multiple nodules in both lungs, aortic and coronary artery calcifications, liver cirrhosis, and bilateral renal cysts. Meanwhile, abdominal ultrasound revealed displayed bilateral renal cysts and prostatic hyperplasia. Vascular ultrasonography revealed lower extremity arteriosclerosis and bilateral carotid atherosclerosis with plaque formation. At the same time, thyroid ultrasonography showed bilateral thyroid nodules. Routine electrocardiography revealed sinus rhythm with first-degree atrioventricular block. Cranial magnetic resonance imaging delineated cerebral arteriosclerosis, with narrowing and partial occlusion of the M2 segment of the left middle cerebral artery and reduced distal branches.

Upon admission, the patient was initially managed with continuous insulin infusion. Nevertheless, his blood glucose levels remained challenging to control during the daytime and even continued to rise beyond the upper detection limit of the glucometer. On the other hand, the blood glucose level rapidly declined after 22:00, with hypoglycemia observed at 04:00, the lowest blood glucose level being 3.6 mmol/L. Subsequently, the patient was managed with intravenous insulin and insulin degludec/liraglutide injection, but neither of them stabilized the alternating episodes of hypoglycemia and hyperglycemia. Given that chronic renal failure can delay insulin clearance, altered insulin pharmacokinetics was initially considered. However, the patient’s significant diurnal fluctuations: the largest amplitude of glycemic excursions (LAGE) was 28.7 mmol/L and the standard deviation of blood glucose (SDBG) was 8.44 mmol/L, characterized by persistent daytime hyperglycemia and spontaneous nocturnal hypoglycemia, as well as the lack of response to several insulin regimens (intravenous insulin, insulin pump, and insulin degludec/liraglutide), were inconsistent with the typical insulin resistance associated with renal disease. Therefore, EIAS, insulinoma, and medication-induced glucose fluctuation were included in the differential diagnosis. A review of the patient’s prescription history allowed the exclusion of drug interactions, and abdominal imaging did not show evidence of insulinoma. Thus, autoantibody profiles were assessed using the YHLO iFlash 3000-H chemiluminescence immunoassay (**Table 1**), whilst insulin and C-peptide levels were quantified using the Alinity i chemiluminescence immunoassay (**Table 2**). Interestingly, the results uncovered markedly high serum insulin levels and high IAA titers, accompanied by a dissociation between serum insulin and C-peptide levels. According to a recent study (6), in some cases of type 2 diabetes with EIAS, blood glucose levels at disease onset are comparable to or marginally above 3.0 mmol/L, in contrast to typical IAS, which generally requires blood glucose levels <2.8 mmol/L. Consequently, a diagnosis of EIAS was made, and insulin was discontinued. As anticipated, no hypoglycemic episodes were observed, although hyperglycemia persisted. Next, a hypoglycemic regimen consisting of 1.2 mg of liraglutide injection subcutaneously once daily, 75 mg of oral dorzagliatin twice daily, and 0.2 mg of oral voglibose tablets three times daily was initiated. Blood glucose levels in the patient steadily stabilized, ranging from 4.9 to 20.2 mmol/L, without episodes of hypoglycemia or excessive hyperglycemia. Serum insulin and IAA levels steadily decreased throughout the follow-up period, whereas blood glucose levels remained steady, ranging from 6.8 to 17.6 mmol/L (**Tables 1**, **2**).

**Table 1:** Autoimmune diabetes antibody profile.

Project	First time	After 10 days	After 60 days	Reference value
Anti-glutamic acid decarboxylase antibody	0.5	0.5	0.5	< 10.0 IU/ml
Insulin autoantibodies	134.6	116.9	52.2	< 1.1 COI
Tyrosine phosphatase antibody	18.3	19.6	19.4	< 10.0 IU/ml
Islet cell antibody	1.9	1.9	1.8	< 1.1 COI

**Table 2:** Blood glucose, C-peptide, and insulin secretion of the first patient.

Project	First time	After 10 days	After 20 days	After 60 days	Reference value
Blood glucose	9.48	3.14	6.94	8.98	3.9–6.1 mmol/L
Insulin	> 600.00	> 600.00	521.30	134.00	1.90–23.00 μIU/ml
C-peptide	7.73	7.12	7.73	2.58	0.78–5.19 ng/ml
Insulin/C-peptide molar ratio	> 10.8	> 11.7	9.3	7.2	mmoL/mmoL

## Case 2

An 86-year-old male patient had a 20-year history of type 2 diabetes and a 10-year history of DKD. He was previously treated with unspecified oral hypoglycemic medications and was then initiated on premixed protamine zinc recombinant human insulin lispro injection (25R) three years ago, administered at 20 units before breakfast and 20 units before dinner. Four months prior to admission, the regimen was changed to insulin glargine (16 units at night) and insulin aspart (6 units in the morning, 6 units at midday, and 5 units at night). Each insulin injection needle was used ten times by the patient. Upon admission, physical examination revealed a temperature of 36.8°C, a pulse of 76 beats per minute, and blood pressure of 123/76 mmHg. Additionally, no abnormalities were identified on cardiopulmonary and abdominal examinations. His past medical history included prostatic hyperplasia, hypertension, chronic renal failure, and long-term routine hemodialysis. He was taking tamsulosin sustained-release capsules, finasteride tablets, and amlodipine tablets.

Laboratory tests demonstrated an FBG level of 11.29 mmol/L, a serum creatinine level of 757 μmol/L, an eGFR of 5.26 ml/(min/1.73 m²), an albumin level of 27.2 g/L, and an HbA1c level of 11.9%. The antinuclear antibody spectrum, thyroid function, and electrolyte levels were within normal limits. Routine scans of the head, abdomen, and chest revealed bilateral renal atrophy, liver cirrhosis, aortic and coronary artery calcifications, and cardiomegaly. Routine ECG assessment revealed a complete right bundle branch block and sinus rhythm. Lastly, echocardiography revealed left artrial enlargement and impaired left ventricular diastolic function.

Since admission, significant fluctuations in the patient’s blood glucose levels were noted (LAGE was 26.9 mmol/L and SDBG was 7.43 mmol/L), with a sharp decline at night and persistently elevated daytime levels peaking at 29.0 mmol/L. Hypoglycemic episodes occurred between 2:00 and 6:00, with the lowest blood glucose reading being 2.7 mmol/L. Chronic renal failure and liver cirrhosis were initially considered the primary causes of glucose fluctuations, given that both conditions impair insulin metabolism and hepatic glucose output (7, 8). Consequently, the dosage of insulin aspart was increased, whereas that of insulin glargine was progressively decreased. Additionally, oral linagliptin tablets (5 mg once daily) were administered. Nevertheless, the patient still experienced fasting hypoglycemia and postprandial hyperglycemia. Thereafter, autoimmune diabetes antibody profiles were analyzed, and insulin and C-peptide levels were quantified (**Table 3**). The results revealed markedly high serum insulin levels and high IAA titers, along with a dissociation phenomenon between serum insulin and C-peptide. As a result, the patient was diagnosed with EIAS, and insulin was discontinued. A hypoglycemic regimen of linagliptin tablets, 5 mg orally once daily, combined with dorzagliatin, 75 mg orally twice daily, was initiated. As anticipated, overall glucose fluctuation improved. Nonetheless, episodes of hypoglycemia persisted. Linagliptin was discontinued, and oral acarbose tablets were added at a dosage of 50 mg three times daily. This adjustment stabilized glycemic control, with blood glucose levels ranging from 6 to 15 mmol/L. Serial monitoring during follow-up demonstrated a progressive decrease in serum insulin and IAA levels (**Table 3**).

**Table 3:** The second patient’s autoimmune diabetes antibody profile and the blood glucose, C-peptide, and insulin secretion table.

Project	First time	After 7 days	After 21 days	After 42 days	Reference value
Anti-glutamic acid decarboxylase antibody	62.0	57.3	50.3	44.9	< 10.0 IU/ml
Insulin autoantibodies	251.7	244.6	199.2	159.2	< 1.1 COI
Tyrosine phosphatase antibody	< 0.7	< 0.7	< 0.7	< 0.7	< 10.0 IU/ml
Islet cell antibody	0.3	0.3	0.4	0.4	< 1.1COI
Blood glucose	2.79	5.67	6.31	7.21	3.9–6.1 mmol/L
Insulin	> 600.00	541.00	171.00	90.90	1.90–23.00 μIU/ml
C-peptide	3.26	8.82	6.22	5.69	0.78–5.19 ng/ml
Insulin/C-peptide molar ratio	> 25.5	8.5	3.8	2.2	mmol/mmol

## Discussion

IAS is an uncommon clinical condition characterized by excessive endogenous insulin secretion and increased IAA levels (9). Human leukocyte antigen (HLA) has been directly associated with its onset, with HLA-RB1*0406, DRB1*0403, DQB1*0302, DQA1*0301, DRB1*0415, and DRB1*130 being potential susceptibility HLA genotypes (10, 11). Among them, HLA-DRB1*0406 is the most prevalent, notably among Asian populations, with the highest frequency identified in Japan. Additionally, IAS exhibits genetic susceptibility and frequently occurs in individuals with comorbid autoimmune diseases. In a subset of cases, it may represent one of the manifestations of autoimmune polyglandular syndrome (APS) (12), with the most common association in Graves’ disease (13). Infections and medications are the two chief causes of IAS. The most prevalent culprits are agents that contain sulfhydryl or sulfhydryl metabolites, including methimazole, propylthiouracil, lipoic acid, imipenem, glutathione, captopril, sulfapyridine, clopidogrel, pantoprazole, rabeprazole, levofloxacin, and isoniazid (14), with methimazole being the most commonly reported (15). Sulfhydryl groups can bind to the disulfide bonds of endogenous insulin, altering its molecular conformation and immunogenicity and triggering the synthesis of IAA. At present, the diagnosis of IAS requires the following conditions: high hyperinsulinemic hypoglycemia, with significantly elevated insulin levels (usually higher than 100 μIU/ml); blood glucose levels below 2.8 mmol/L; elevated IAA levels; and the absence of exogenous insulin administration (16).

EIAS is a non-classical form of IAS, caused by exposure to exogenous insulin. It is characterized by hyperinsulinemia, the presence of positive insulin antibodies, hyperglycemia, hypoglycemia, or alternative episodes of both following exogenous insulin administration. To date, no standardized diagnostic criteria are available for EIAS. Therefore, we propose that the following details be included in the diagnosis of EIAS: (1) History of exogenous insulin use, accompanied by unexplained glycemic fluctuations (hypoglycemia defined as <3.9 mmol/L in diabetic patients, <2.8 mmol/L in non-diabetic patients, combined with hyperglycemia); (2) Persistent hypoglycemia in diabetic patients despite reduction or discontinuation of insulin therapy; (3) Hyperinsulinemia (insulin >100 μIU/ml); (4) An insulin-to-C-peptide molar ratio (ICPR) >1. Based on the above diagnostic criteria, the EIAS diagnosis was confirmed in both patients. In contrast to classical IAS, the most significant challenge in identifying EIAS is the impact of exogenous insulin on serum insulin level measurements and the inability to measure C-peptide levels in patients with pancreatic β-cell dysfunction. Exogenous insulin administration generally results in high blood insulin levels and C-peptide levels near 0 ng/ml, which may lead to a high false-positive rate when using the ICPR >1 criterion. There is currently no definite or reliable approach to address these challenges. Furthermore, in the two cases, in addition to positive IAA, patient 1 was positive for IA-2, while patient 2 was positive for GADA, findings that are exceedingly rare in EIAS patients. IA-2 and GADA are typically regarded as markers of LADA. However, an earlier study (17) has identified a potential overlap between EIAS and LADA in terms of autoimmune susceptibility. In other words, an autoimmune predisposition may serve as both a risk factor for EIAS and the development of LADA. Follow-up of the two patients revealed that C-peptide levels did not decrease as rapidly as observed in LADA. Therefore, the possibility of concurrent LADA cannot be excluded. To confirm the diagnosis, long-term monitoring of C-peptide and autoantibody levels is necessary.

Noteworthily, nearly all types of insulin have been implicated in EIAS (18), with premixed insulin formulations accounting for around 62% of cases (19). The IAA induced by EIAS is hallmarked by high affinity and low capacity, indicating extensive and non-reversible insulin binding, eventually culminating in insulin resistance and hyperglycemia. Besides, it can also induce spontaneous hypoglycemia (4). In diabetic patients with EIAS, the hypoglycemia threshold may be adjusted to <3.9 mmol/L, consistent with the definition of diabetic hypoglycemia, due to their baseline impaired glucose metabolism (6). Furthermore, a dissociation between serum insulin and C-peptide levels is commonly observed, signifying that increasing serum insulin levels are disproportionate to alterations in C-peptide levels. A previous study has concluded that in individuals receiving exogenous insulin, the presence of insulin antibodies may result in EIAS if the insulin-to-C-peptide ratio in the fasting state surpasses 8.6 or 17.8 2h postprandially (14). More importantly, recent research indicates that EIAS exhibits greater glycemic variability in clinical settings, which is related to ICPR. Genetically, EIAS is linked to a unique susceptible HLA haplotype (e.g., DRB10405-DQA103-DQB1*0401), which differs from type 1 diabetes or immune-mediated diabetes. Proteomic analyses have identified nine differentially expressed proteins (DEPs), among which GALNT3, IL10, and CCL28 hold diagnostic potential, reflecting enhanced immune-mediated inflammation (20).

The mechanisms by which exogenous insulin causes EIAS remain elusive, although they are partially mediated by IAA. The amino acid sequence of insulin analogues is different from that of endogenous human insulin, allowing them to be considered non-self antigens. Zinc-mediated insulin can persist longer at the injection site and be more readily taken up by antigen-presenting cells (2). In addition, hyperplasia of subcutaneous fat at the insulin injection site prolongs insulin retention and delays insulin release (21), increasing the risk of IAA formation and an immune response. It is worthwhile emphasizing that hyperplasia of subcutaneous fat is directly associated with the frequency of injection needle reuse, with repeated needle use increasing the risk of IAA formation (22). Insulin needles were not frequently changed in these two patients, and the first patient exhibited several regions of abdominal subcutaneous fat hyperplasia, which might be connected to the development of EIAS.

Both patients had DKD as the primary cause of chronic renal failure. Chronic renal failure plays a key role in exacerbating EIAS symptoms. Chronic renal failure impairs gluconeogenic pathways, reduces renal clearance of insulin, impairs insulin degradation due to uremia, increases glucose uptake by red blood cells during hemodialysis, and diminishes counter-regulatory hormonal responses (23, 24). These factors collectively contributed to the severe and prolonged glycemic dysfunction in both patients. EIAS-induced erratic glucose fluctuations may further damage renal tubules and glomeruli, thereby accelerating DKD progression. Recurrent hypoglycemia lowers the renal energy supply, while hyperglycemia increases oxidative stress and the buildup of advanced glycation end products (AGEs) in renal cells, further harming tubules and glomeruli (25).

The alternating hypoglycemic and hyperglycemic episodes of EIAS can impact cardiac function. Hypoglycemia in diabetic patients damages potassium ion channels, induces hypokalemia and catecholamine release, delays repolarization, activates the sympathetic nervous system, and limits myocardial energy supply, thereby increasing the risk of arrhythmias, such as QT prolongation (26). Furthermore, hyperglycemia is also detrimental to cardiovascular health (27). Indeed, both patients had abnormal ECG findings, including atrioventricular block and right bundle branch block, although no abnormalities in QT intervals were observed. Quickly correcting hypoglycemia, avoiding grade 2 hypoglycemia (<3.0 mmol/L), CGM and TIR evaluation, and close observation of QT intervals in CVD and CKD patients can effectively mitigate the impact of hypoglycemia on the cardiovascular system (28). Furthermore, in cases of hypoglycemia, administration of potassium supplements or beta-blockers can alleviate mild arrhythmias (29).

Currently, there is no standardized treatment protocol for EIAS. The primary treatment goals are to eliminate IAA, correct and prevent hypoglycemia, and provide symptomatic and etiological interventions. Symptomatic management largely involves dietary interventions, optimizing meal composition, avoiding strenuous and fasting exercises, and engaging in light exercise after meals (30). In addition, oral α-glucosidase inhibitors can be prescribed to lower postprandial hyperglycemia (31). In contrast, etiological treatments include discontinuing exogenous insulin and the administration of immunosuppressants, such as glucocorticoids, mycophenolate mofetil, rituximab, and so forth (32–35). Furthermore, plasma exchange may be taken into consideration if immunosuppressive therapy is insufficient (36). Upon cessation of exogenous insulin, the two individuals described in this article experienced relief from nocturnal and fasting hypoglycemia; thus, hormone therapy was not initiated. Nevertheless, hyperglycemia remained unabated. These two patients experienced severe renal impairment, including progression to end-stage renal disease, which restricted available hypoglycemic options. Additionally, a combination of dorzagliatin and α-glucosidase inhibitors was used in both individuals. According to prior investigations, dorzagliatin, a glucose kinase activator, has a renal clearance rate of less than 10% of the administered dose, rendering it safe in patients with renal failure (37). It can also enhance glucose kinase function, reduce blood glucose levels by driving the synthesis of insulin under hyperglycemic conditions, and raise blood glucose levels by promoting the production of glucagon under hypoglycemic conditions (38). Consequently, it can improve glycemic control in EIAS patients. In addition, the follow-up data revealed that both patients’ ICPR had dramatically dropped from the starting levels, suggesting that the EIAS condition had improved.

## Conclusion

EIAS is a rare condition with currently no established diagnostic criteria or treatment strategies, and its pathophysiology remains to be elucidated. This article reports two cases of EIAS complicated by chronic renal failure and proposes critical diagnostic considerations. Further evidence-based studies are warranted to validate the therapeutic effects of dorzagliatin.

## Data Availability

The original contributions presented in the study are included in the article/**Supplementary Material**. Further inquiries can be directed to the corresponding author.
