# Supplementary figures and images for: Case Report: Exogenous insulin antibody syndrome complicated with chronic renal failure and long-term history of type 2 diabetes: report of two cases
Source: Front Endocrinol (Lausanne). 2025 Oct 29;16:1676062. doi: 10.3389/fendo.2025.1676062 (PMC12605513; doi:10.3389/fendo.2025.1676062)

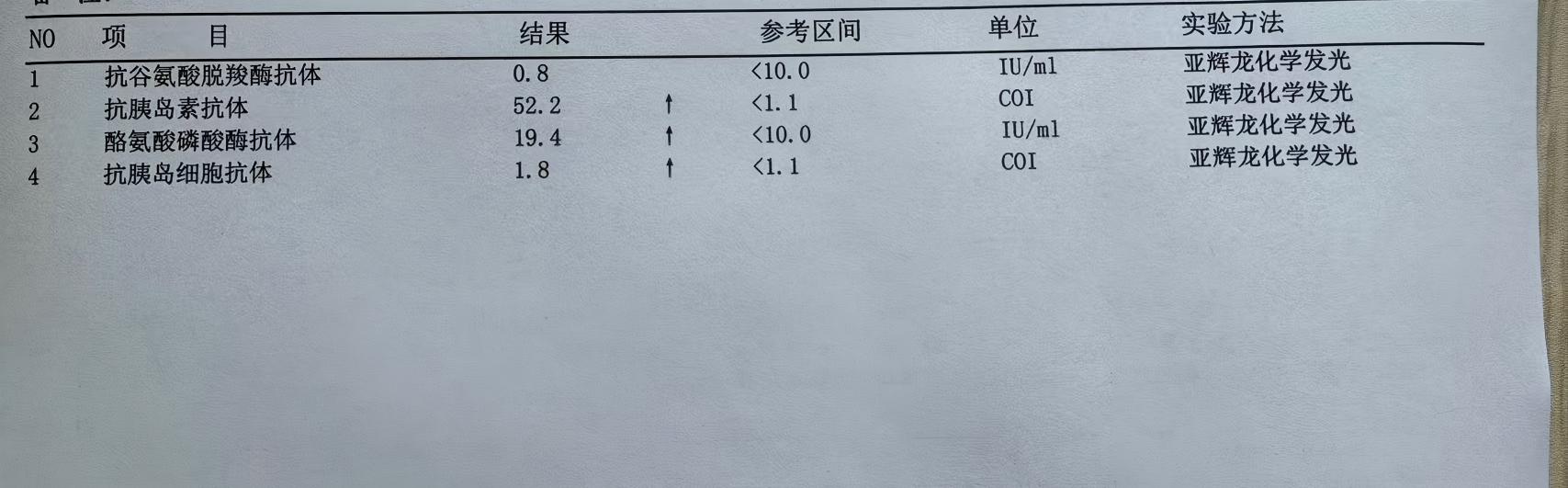

Supplement: Supplementary file 1 [file Image1.jpeg]
