# Supplementary material for: Case Report: Exogenous insulin antibody syndrome complicated with chronic renal failure and long-term history of type 2 diabetes: report of two cases
Source: Front Endocrinol (Lausanne). 2025 Oct 29;16:1676062. doi: 10.3389/fendo.2025.1676062 (PMC12605513; doi:10.3389/fendo.2025.1676062)
Supplement: Supplementary file 2 [file DataSheet1.pdf]

Supplementary Table1 Blood glucose measurements at various time points during hospitalization of the first patient(mmol/L)

Time Point	Day1	Day2	Day3	Day4	Day5	Day6
6:00	-	3.6	3.9	4.1	5.4	5.2
8:00	20.3	11.8	12.6	14.6	13.8	11.7
16:00	23.6	25.4	16.9	16.7	14.9	-
18:00	29.8	26.8	19.7	20.3	17.4	-
22:00	32.3	30.3	23.7	22.8	19.3	-

(-,The patient did not undergo hospitalization or has already been discharged, thus no monitoring of blood sugar was conducted; LAGE ,largest amplitude of glycemic excursions=28.7 mmol/L; SDBG,standard deviation of blood glucose=8.44 mmol/L)

Supplementary Table2 Blood glucose measurements at various time points during hospitalization of the second patient(mmol/L)

Time Point	Day1	Day2	Day3	Day4	Day5	Day6	Day7
6:00	-	2.7	3.5	4.1	4.4	4.9	5.2
8:00	14.8	16.8	15.9	13.6	15.6	14.7	12.6
16:00	19.6	20.2	17.3	16.8	18.3	16.3	-
18:00	25.7	23.1	20.2	20.2	22.3	19.7	-
22:00	29.6	28.8	26.9	20.6	21.6	18.9	-

(-,The patient did not undergo hospitalization or has already been discharged, thus no monitoring of blood sugar was conducted; LAGE=26.9 mmol/L;SDBG=7.43 mmol/L)
